# Fifteen Years of Regulating Nutrition and Health Claims in Europe: The Past, the Present and the Future

**DOI:** 10.3390/nu13051725

**Published:** 2021-05-19

**Authors:** Alie de Boer

**Affiliations:** Food Claims Centre Venlo, Campus Venlo, Faculty of Science and Engineering, Maastricht University, 5900 AA Venlo, The Netherlands; a.deboer@maastrichtuniversity.nl

**Keywords:** European Food Safety Authority, nutrition, functional foods, European food law, risk assessment

## Abstract

Suggestions that a food contains healthy ingredients or that it can provide beneficial effects upon consumption have been regulated in the EU since 2006. This paper describes the analysis of how this nutrition and health claim regulation has resulted in over 300 authorised claims and how the authorisation requirements and processes have affected the use of claims on foods. Five challenges are identified that negatively affect the current legislation dealing with nutrition and health claims: non-reviewed botanical claims (as well as on hold claims for infants and young children), the lack of nutrient profiles and the focus of claims on single ingredients, consumer understanding, research into health effects of nutrition and finally, enforcement. These challenges are shown to influence the goals of the regulation: protecting consumers from false and misleading claims and stimulating the development of a level playing field in the EU, to foster innovation. Tackling these political and scientific substantiation questions for health claims, together with continuously analysing the understanding and usage of claims by consumers and operators will ensure that the NHCR will stay effective, today and in the future.

## 1. Introduction

Labels, policy instruments regulating the presentation of product-specific information to consumers, can play an important role in providing insights into ‘invisible’ or credence attributes of food products [1]. Such attributes that consumers cannot ascertain themselves upon purchase or consumptions but that are of interest to them, can be related to, i.e., production techniques, the effects of the product on the environment or related to potential health benefits of consuming a food [1,2]. Since these credence attributes are not visible or measurable by consumers themselves, public and private organisations aim to reduce information asymmetry between those developing and selling produce and those citizens purchasing them by requiring adequate labelling [3]. When labels truthfully provide this type of information, they can be used by consumers to inform themselves and by producers to share their unique selling points [4]. Less trustworthy labelling, when food business operators mislead consumers with deliberate false information, is not considered in this paper. Still, also truthful labelling has been described as having limitations: literature, e.g., describes that it does not necessarily improve the position of consumers nor welfare due to price competition, and that it can negatively affect strategic choices of suppliers [1]. Still, labelling should offer the opportunity for producers of high-quality products to provide full information and increase consumers’ willingness to pay [1].

In Europe, food information is considered to allow consumers to get appropriately informed about their foods, as laid down in the European Regulation on food information to consumers (FIC), Regulation (EU) No 1169/2011 [5]. The FIC Regulation defines various elements that are mandatory for inclusion on labels, as described in recital 17 of the regulation ‘to enable consumers to identify and make appropriate use of a food and to make choices that suit their individual dietary needs’ [5]. Whereas this regulation deals with food information in general, information about nutrition and health effects of foods are regulated separately. Since December 2006, statements made on foods about their beneficial ingredients or beneficial effects, are regulated in the European Union. Almost 15 years ago, Regulation (EC) No 1924/2006 on nutrition and health claims entered into force, which aimed to protect consumers from false and misleading claims and prohibited medical claims on foods [6]. The EU was not the first jurisdiction to regulate statements related to nutritional content or implied health effects of foods or food ingredients: already in 1991, the ‘foods for specified health uses’ (FOSHU) system was developed in Japan [7] and other countries followed in the years thereafter, including but not limited to Australia and New Zealand, Brazil, Canada, China, Mexico, the Republic of Korea and the United States of America [8]. Even though globally, similar terminology is used to define these types of claims, regulatory differences exist in what claims are allowed on food products or other categories of health enhancing products, and what type of evidence is required to support these claims [8,9]. In many countries, claims need to be based on scientific evidence, but in Europe specifically, nutrition and health claims need to be authorised prior to their usage on the market. Scientific evidence is key in this authorisation decision [8,10].

Developing legislation to deal with nutrition and health claims on foods in the EU was one of the 84 action points defined in the extensive reform of food law, in response to EU-wide food scares in the 1990s [11,12]. Since 2002, the General Food Law (GFL, Regulation (EC) No 178/2002) lays the foundation for food law, regulating food, food safety and food production ‘from farm to fork’ [13,14]. With this regulation describing the basic requirements for foods, more detailed legislation followed to regulate specific aspects in the food chain, including, i.e., food additives and labelling of food products [5,15]. The Nutrition and Health Claim Regulation (abbreviated as NHCR) is similarly dedicated to dealing with a distinct type of food information: voluntary messages that can be used to promote foods as containing nutritionally beneficial ingredients or products that have a favourable effect on health. In 2013, the evaluation of the legislative framework for foods was announced in the European Commission’s Fitness Check, known as the Regulatory Fitness and Performance Programme (REFIT) [16]. To review whether the current legislation was still deemed ‘fit for purpose’ in dealing with current and future food safety and food policy measures, such an evaluation was started for the NHCR in 2015 [17,18,19]. The evaluation that was concluded in 2020, highlighted that currently, the objectives of the NHCR are not fully attained [20]. Not all types of claims on foods have been reviewed: claims on herbs and herbal preparations (known as botanical claims) that have a long history of use for specific health benefits have not been assessed yet. In addition, without the development of nutrient profiles, health claims can still be used on products that exceed thresholds of specific nutrients, such as fat, sugars and salt [20].

Over these last fifteen years, nutritional sciences and the use of nutrition in maintaining and improving health have continued to develop [21]. Consumers are increasingly interested in health when making decisions related to their diet: in using nutrition to maintain their health and preventing diseases, and even in managing diseases which they are already suffering from [21,22,23]. At the same time, health in general and healthy foods specifically have gained relevance amongst consumers [24,25,26]. This has led to increased popularity of specific products that are promoted for their health benefits (such as dietary supplements), especially amongst those consumers who have already adopted a healthier lifestyle [27,28,29]. Are the recommended suggestions to improve the effectiveness of the NHCR—dealing with botanicals and adopting nutrient profiles—sufficient to deal with these ongoing developments? In this paper, the development and use of the nutrition and health claim regulation over the last 15 years will be analysed and an outlook is provided on the potential of the regulation to deal with nutritional sciences and consumer health interests in the upcoming 15 years. For that purpose, the current state of (non-)authorised nutrition and health claims in the EU has been studied from an explorative, interdisciplinary perspective. Through this conceptual approach [30], insights from regulatory, food policy, nutrition and consumer sciences were brought together, providing multi-level insights into the topic of nutrition and health claims. It highlights specific aspects that require further attention to ensure that nutrition and health claims can contribute to adequately protecting consumers from misleading information. This study thereby allows for broadening the theoretical and practical understanding of claims, as well as obstacles and opportunities in improving their regulation.

## 2. Nutrition and Health Claims in Europe in the Past 15 Years

The European Nutrition and Health Claim Regulation was preceded by various research projects and policy documents providing insights into how to deal with advertisement and information to consumers about functional foods [31]. The 1995 Functional Food Science (FUFOSE) project and the 2001 project Process for Assessment of Scientific Support for Claims on Foods (PASSCLAIM) suggested that specific categories should be developed for claims on foods and provided criteria for their scientific substantiation [32,33]. At the same time, in 2000, the European Commission described their intention to regulate nutrition and health claims in the White Paper on Food Safety [12]. Via various policy documents and proposals that were informed by the research projects, the final regulation was adopted in December 2006 [31].

With the NHCR, consumers should be offered the highest level of protection from misleading by false, ambiguous or misleading statements, as well as from medical claims made on foods [6]. Claims are defined as statements that refer to either the nutritional content of a food (nutrition claims) or those referring to health benefits of specific nutrients, ingredients or foods (health benefits). As described in Article 3 of the Regulation, the fact that claims cannot be misleading also entails that statements may not raise doubts about the safety or nutritional adequacy of other food products, and that they cannot be used to suggest that a varied and balanced diet would be unable to provide sufficient quantities of nutrients. In the definition of the NHCR, claims go beyond just textual statements found on food labels: they are defined as any voluntary message that suggests that *‘food[s have] particular characteristics’*, including in the form of pictures, graphics or symbols. Even trademarks, brand names or fancy names that suggest that foods have beneficial characteristics should be accompanied with authorised claims [6]. As displayed in Table 1, 30 nutrition claims and 265 health claims are currently authorised in the EU whilst 44 claims await a decision by the risk manager (Table 2).

### 2.1. Nutrition Claims

All claims that are authorised for use on food products in the EU are placed on a positive list of authorised claims. For nutrition claims, this positive list can be found in the Annex of Regulation (EC) No 1924/2006. Today, 30 nutrition claims are authorised within the EU market, relating to statements that suggest or imply that a food has specific beneficial nutritional properties. These claims describe the nutrients or energy that a product (a) contains or provides, (b) contains or provides in increased proportions, (c) contains or provides in reduced proportions or (d) does not contain [6]. Those statements that are not literally found on the positive list, but have a similar meaning for a consumer, fall into the scope of nutrition claims and thus need to comply with the defined conditions of use. An example described in the recitals of the NHCR is the use of the term ‘enriched’ when referring to a specific nutrient, which is considered to have the same meaning as the ‘source of’ claim that is defined in the positive list. For both these claims, it is important that the vitamin or mineral that is the subject of the claim can be found in the product in a ‘significant amount’, providing at least 15% of the recommended dietary intake of that nutrient [6].

Nutrition claims are based on national or international agreements regarding the fact that the nutrient is beneficial, or that the removal (or non-addition) of such a nutrient from food is considered beneficial. Nutrition claims can be used on all types of foods, only for alcoholic beverages containing more than 1.2% volume of alcohol, specific requirements are made: only claims may be used that refer to low alcohol levels, or reduced alcohol or energy content. However, as defined in Article 4 of the NHCR, MS legislation may set stricter rules for (not) allowing these types of claims [6].

New nutrition claims can be added to the positive list laid down in the Annex through newly proposed Commission Regulations, by the European Commission. These proposals are developed based on consultations with different stakeholders (including consumer groups, food business operators and Member States (MS)). Whereas the Annex initially included 24 nutrition claims when the Regulation entered into force in 2006, 5 additional claims related to unsaturated fatty acids were authorised in 2010 and finally in 2012, the nutrition claim ‘no added sodium/salt’ was added to the positive list [34,35]. This brings the total of authorised claims to 30, all described in the Annex of the most recent consolidated version of the NHCR, of 13 December 2014.

### 2.2. Health Claims

Whereas nutrition claims are statements referring to the nutritional content of a product, health claims are statements that go one step further: they link a specific food (ingredient) to a health benefit, implying that consuming such a product supports health of the consumer. Within the NHCR, four types of claims have been distinguished: (i) function claims based on generally accepted scientific evidence (Article 13.1); (ii) function claims based on newly developed scientific insights (Article 13.5); claims referring to reducing a risk factor in disease development (Article 14.1(a)); and claims referring to growth and development of children (Article 14.1(b)) [6]. All types of claims must be substantiated with scientific evidence to support the causal relationship between the intake of the ingredient and the health benefit described in the claim.

#### 2.2.1. Scientific Substantiation

To support the causal relationship between consuming a food (ingredient) and the consequential beneficial physiological effect, evidence needs to be provided that a *healthy population* benefits from the ingredient [6]. Referring to disease prevention, treatment or even curing such a disease is not allowed: this presentation would result in the product that carries the claim being considered as a medicinal product instead of a food [5,36,37]. After receiving the authorisation request and having reviewed the eligibility of the claim, the European Commission asks the European Food Safety Authority (EFSA) to independently review the scientific evidence that has been provided by the food business operator applying for the claim. The dossier submitted by the applicant must contain information on this causal effect, in which three elements are key: (i) the ingredient needs to be well characterised; (ii) the well-defined effect needs to be beneficial and needs to be established in a healthy population; and (iii) the causal link must be substantiated with well designed (randomized, double blind, controlled) trials in humans [38,39]. As defined in Commission Regulation (EC) No 353/2008 as well as EFSA’s general guidance document to support health claim applications, it must also be shown that the quantity and consumption pattern for the ingredient—to obtain the health benefit—is achievable within a balanced diet [39,40]. 

The independent experts within the EFSA NDA Panel (the EFSA Panel on Nutrition, Novel Foods and Food Allergens) will review whether all available scientific evidence has been considered in the application. The Panel can request additional information from applicants in case of unclarities in the dossier. Next to the general guidance document, with the assessment of Article 13.1 claims having finished and after reviewing various requests for Article 13.5 and Article 14.1 claims, the NDA Panel has published additional guidance documents to provide insights into the scientific requirements related to specific types of health outcomes. This includes guidance on health claims related to antioxidants, oxidative damage and cardiovascular health [41]; bone, joints, skin and oral health [42]; appetite ratings, weight management and blood glucose concentrations [43]; functions of the nervous system, including psychological functions [44]; physical performance; and the immune system, the gastrointestinal tract and defence against pathogenic microorganisms [45]. These guidance documents should support food business operators in preparing their scientific dossiers, since many putative health claims were considered insufficiently substantiated with scientific evidence to support the proposed health relationship [38,46]. 

Whereas there is no amount of studies pre-defined needed to support a health claim, providing at least two independently conducted human intervention studies of high quality to support the causal relationship between consumption and beneficial effect is often deemed necessary [38,47]. When all available evidence is considered supportive of the proposed claim, the EFSA NDA Panel will give their positive opinion about the claim and send this to the European Commission. This opinion may include specific conditions of use (related to the quantity and consumption pattern), as well as comments on the proposed wording of the claim, to ensure that the scientifically substantiated relationship is well reflected in the claim.

#### 2.2.2. Authorisation Procedure

**Figure 1:**
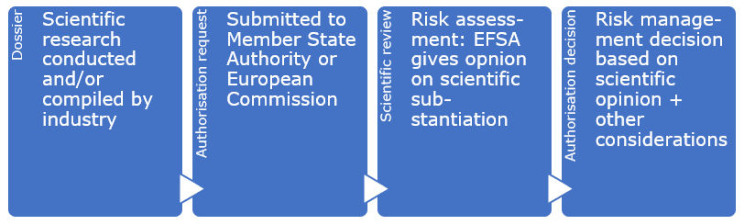
General process for authorisation requests for food and nutrition.

Authorisation requests for health claims are dealt with in two ways: firstly, function claims based on generally accepted scientific evidence needed to be submitted by 1 January 2008 [6]. All submitted claims were grouped and subsequently sent to EFSA for assessing whether the supportive evidence—that could be based on consensus documents and expert reports—indeed supported the suggested health benefit. However, new additions to the list of function claims (Article 13.5 claims) as well as individual applications under Article 14.1(a) and Article 14.1(b) follow a second, different procedure. Similar to other food authorisation procedures in the EU [48], a food business operator thus submits the evidence with the authorisation request to the European Commission (Figure 1). The Commission can ask EFSA to review the scientific evidence supporting the putative claim, who will provide their scientific opinion about the claim. Their scientific opinion is key in the Commission’s decision to authorise a claim when they submit their draft decision for authorisation to the Standing Committee on the Food Chain and Animal Health, the regular procedure for authorising new claims. As described in Article 17 of the NHCR, there may be cases where the Commission deviates from EFSA’s opinion, because of ‘relevant provisions from EU legislation’ or other ‘legitimate factors’ [6].

The Commission is seen to follow EFSA’s scientific opinions closely, but in few cases for which the evidence was described to support a cause-and-effect relationship, have not been authorised on the European market: five claims on glucose supporting the human metabolism were considered inconsistent with generally accepted principles in nutrition and health [49,50]. The Commission’s decision was challenged by the food business operator but upheld by the General Court [51] and the subsequent appeal of the food business operator was dismissed by the Court of Justice of the European Union in 2017 [52]. In addition, four claims referring to increased alertness from caffeine consumption were by EFSA considered to be sufficiently supported by scientific evidence, establishing a cause-and-effect relationship [53]. Whereas the Commission proposed to authorise these claims, Members of the European Parliament rejected the claims because of public health concerns, related to the potential effect of allowing caffeine claims on drinks targeted to adolescence or drinks containing high amounts of sugar [54]. As displayed in Table 2, 44 health claims upon which a scientific opinion has been issued, await an authorisation decision from the Commission today.

#### 2.2.3. From Submission to (Non-)Authorisation

With over 44,000 Article 13.1 claim authorisation requests submitted by 2008 [31], a total of 229 Article 13.1 health claims is authorised today. The first 222 authorised (Article 13.1) health claims were placed on the positive list, the list of permitted health claims made on foods, other than those referring to the reduction of disease risk and to children’s development and health in Regulation (EU) No 432/2012 [55]. The positive list only contains Article 13.1 and 13.5 claims, since Article 14.1(a) and 14.1(b) claim applications are individual authorisation requests for which the authorisation decision is published in separate legislative acts. No new submission can be filed for Article 13.1 claims, but there have been various additions and amendments of both Article 13.1 and Article 13.5 claims to the list after December 2012. Seven claims were added to the list in 2013 and 2014 because their scientific evaluation had not been finished before Regulation 432/2012 was drafted. These claims related to health benefits associated with alpha-cyclodextrin, fructose, dried plums, docosahexaenoic acid (DHA) and twice with a combination of DHA and EPA (eicosapentaenoic acid) [56]; as well as one claim describing the link between carbohydrate and maintenance of normal brain function [57]. In 2016, adjustments were necessary to the conditions of use for claims on meal replacing products for weight control, due to legislative changes to this category of products under Regulation (EU) No 6092/013, the regulation that deals with foods for special groups [58]. The 229 claims authorised today—often dealing with vitamins and minerals—all refer to health effects that describe the function of an ingredient in physiological processes. They describe either how a food (ingredient) contributes to maintenance of a function, enhancement of a function or how it supports homeostasis, for example describing that calcium contributes to normal muscle function. As compiled since 2012, there are 2078 botanical claims (with 2133 original ID numbers) still pending finalisation [59,60]. The Commission and the Parliament have not yet decided upon whether these claims need to be reviewed similarly to other food related claims, or whether different substantiation requirements should be defined. Suggestions are made to allow data on ‘traditional use’ to support such claims, which is also allowed for substantiating safety and claims of traditional herbal medicinal products [17,61]. 

Whereas generally accepted scientific evidence, such as consensus documents and workshop statements, can be sufficient for substantiating Article 13.1 claims, Article 13.5 claims need to follow the procedure as described above: specific data must be submitted that supports the beneficial physiological effect of the compound for the general healthy population. This means that whereas for Article 13.1 claims, reference could be made to such consensus documents to support a claim, dossiers supporting Article 13.5 claims need to be more extensive, specific and need to contain detailed information to support the causal relationship between consuming the ingredient and relevant health outcomes, as established by appropriate measurement methods. When applying however for such an Article 13.5 claim, food business operators that gather such data may receive the exclusive right to use a claim for the first five years of its authorisation, when these data are considered proprietary data [6]. After this period of five years, the claim becomes a generic health claim and for other products for which the conditions of use are fulfilled this claim may be used. So far, 11 Article 13.5 claims have been authorised, and for 6 of these claims a protection of proprietary data has been granted. In 2009, the first claim was authorised, describing health effects of water-soluble tomato concentrate [62]. Interestingly, this claim—as well as its 2010 amendment [63]—is not described on the positive list. All 10 other claims are found on this positive list. In 2013, the first three Article 13.5 claims with protection of proprietary data were added to Regulation 432/2012 [64], which was followed in 2014 by another claim (on sugar beet fibre) [65], two health claims on carbohydrates [66] and inulin (with proprietary data protection) [67] and an amendment to the cocoa flavanol claim in 2015 (again with proprietary data protection) [68], and finally, four claims were added in 2016 (on non-fermentable and non-digestible carbohydrates [69]) and 2017 (on creatin [70] and lacitol [71]). Since 2021, all granted data protection has been expired and therefore, any product meeting the conditions of use for the authorised claim can use these claims. Similar to Article 13 claims, the health relationships used in authorised Article 13.5 claims mainly define how a food (ingredient) contributes to maintenance of a function, enhancement of a function or how it supports homeostasis, as exemplified by the authorised claim on non-digestible carbohydrates: Consumption of foods/drinks containing <describe used non-digestible carbohydrates> instead of sugars induces a lower blood glucose rise after their consumption compared to sugar-containing foods/drinks.

Similar to all nutrition claims and function claims described above, also individually authorised claims under Article 14 (disease risk reduction claims) are generic claims and can thus be used by both the applicant as well as other food businesses producing similar products. The decision upon authorisation or rejection of these claims is published in dedicated Commission Regulations and information on these claims (e.g., their conditions of use in case of authorised claims) can be accessed through the EU’s Register of Health Claims (found on https://ec.europa.eu/food/safety/labelling_nutrition/claims/register/, accessed on 16 March 2021). The Register currently includes information upon 41 claim authorisation requests for claims referring to the effect that a food (ingredient) has on a risk factor in disease development (Article 14.1(a) claims), and 57 entries for requesting authorisation for Article 14.1(b) claims, those claims referring to children’s development and growth. Only finalised authorisation requests can be found in the Register; the 29 submitted pending authorisation decisions (Table 2) are not displayed.

Fourteen disease risk reduction claims have been authorised within the EU. Key in applying these claims is the use of a two-step approach to describe the health effect: firstly, the relationship between a food (ingredient) and its effect on a risk factor is stated, followed by linking this risk factor to disease development. This two-step approach is needed to ensure that consumers can see that this risk factor is not the only factor influencing disease occurrence, contributing to protecting consumers from being misled [72]. Most authorised disease risk reduction claims deal with blood cholesterol lowering effects of either barley beta-glucans, oat beta-glucans, plant sterols and stanol esters, or monounsaturated and/or polyunsaturated fatty acids. The second part of these claims always reads that ‘high cholesterol is a risk factor in the development of coronary heart disease’. Other authorised Article 14.1(a) claims describe the effects of sugar-free chewing gum or chewing gum sweetened with 100% xylitol on plaque acids or tooth demineralisation, vitamin D on reducing the risk of falling, calcium (also in combination with vitamin D) on bone mineral density and link folic acid to increased maternal folate status. Authorisations were issued between 2009 and 2014.

The final type of claims are statements referring to the effects of food (ingredients) on growth and development of children, Article 14.1(b) claims. Twelve claims have been authorised from 2009 to 2016, including for example the claims that calcium, calcium and vitamin D, phosphorus and protein is needed for normal growth and development of bone in children. All authorised claims describe essential roles of nutrients in growth and development or the supportive effects of substances in growth and development. A large number of claims (25) is however still pending, despite 24 positive scientific opinions issued by EFSA upon the causal relationship that has been demonstrated between consumption and health effects in children. As many of these positive opinions refer to a specific target group, infants and young children up to three years of age, the authorisation or rejection of these claims seems to depend on the risk manager’s decision to (not) allow claims directed to (the parents of) this target group.

### 2.3. Using Authorised Claims

All authorised nutrition and health claims can be used on food products throughout the EU, as there are currently no claims with existing protected data. It is essential that the food product upon which the claim is used, aligns with the conditions of use of a claim. This is true for both nutrition and health claims. However, as soon as these conditions of use are met, it is not necessary for the technical wording in which the claim was approved to be used on a label: as long as claims have similar meanings, they are considered to fall under this authorisation [6]. The flexibility of wording allows food business operators to present a health claim in a more attractive way for marketing purposes, as long as the wording still reflects the health effect as was authorised. Even though national enforcement authorities in MS may differ in their interpretation of the exact flexibility [72], it has become clear that words such as ‘normal’ and ‘maintenance’ are essential to still be well reflected in reworded and translated claims.

Next to this flexibility of wording, authorised health claims also provide the opportunity for food business operators to make use of more general well-being claims, claims that were initially foreseen to be not allowed for use on food products anymore because of the NHCR [73]. When a product meets the conditions of use for an authorised claim, based on Article 10(3) of the NHCR, a more general claim can be made about the product, as long as this claim is accompanied by the authorised Article 13 or 14 claim. Still, the use of terminology in these statements is restricted, as the NHCR only allows the use of general statements that refer to overall good health or health-related well-being [73,74]. Similar to such general statements, also other sources of voluntarily provided information about nutritional content or health effects are considered claims [6,72,75]. This means that also online information, information in advertisement and campaigns, as well as symbols and graphics that describe or imply to influence nutritional intake or health are regulated as claims.

## 3. Today’s Challenges in Substantiating and Using Claims

The NHCR was published 15 years ago and almost 10 years ago, the positive list of claims was issued in which 222 Article 13.1 claims were authorised. As described in the previous section, today 30 nutrition claims and 265 authorised health claims are allowed for use in the EU market. As previously described in literature, many proposed health claims were considered insufficiently substantiated with scientific evidence [31,38,46]. Thereby, false and misleading claims should be removed from the market and food business operators have gained clear insights into their ability to use specific claims on food products. As introduced in the first section of this paper, still a few challenges are known to exist today in the use of authorised claims, as well as the substantiation of health effects for new claim applications. In this section, the five most prominent challenges of regulating claims today will be discussed into more detail: botanical claims, the single ingredient focus, consumer understanding, health effects research and finally, enforcement.

### 3.1. Botanicals

The 2020 REFIT evaluation defined two main causes for the NHCR to currently not completely reach its objectives. Firstly, the claims on botanicals that have not yet been assessed, and secondly the fact that nutrient profiles have not yet been developed [60]. With the entry into force of the NHCR in 2006 and the call to submit dossiers for claims on food products that were based on generally accepted scientific evidence by 2008, many claims were submitted that dealt with health effects of botanicals [46,60]. A number of these dossiers refers to the traditional use of these products for their putative health benefits, a type of information that is also used for traditional herbal medicinal products [61]. Under the current legislative procedure for claims, such data are not considered to sufficiently substantiate health claims. However, various stakeholders call for updating the procedure in such a way that it will be made possible to use these claims [17]. No risk management decision on how to move forward with these claims is expected any time soon. This creates a challenging situation: botanical claims that were submitted before January 2008 can be used on products (in the Netherlands for example accompanied by a disclaimer, that the claim is pending evaluation), even though varieties of these claims are not supported by evidence similar to the scientific insights supporting authorised claims. As displayed in Table 2, this means that over 2000 claims can be used today without a proper scientific assessment having taken place. It is questionable whether consumers, who are faced with these different types of claims, can clearly distinguish these non-reviewed claims from the claims that have been scrutinised upon their scientific merit. In the REFIT evaluation report, it was already pointed out that to ensure the NHCR can become more effective in protecting consumers from false and misleading claims, it is necessary to address this issue on risk management level [60]. This is the only way to ensure that these claims can be well-used by food business operators and consumers for providing and gaining insights into nutrition and health.

### 3.2. Healthy Ingredients Versus Healthy Foods

The second point raised in the NHCR evaluation deals with the lack of developing nutrient profiles for foods: claims may be used on products that do not have a favourable nutrient profile, such as sodas or candy that provide high amounts of unfavourable ingredients (such as fat, sugars and salt) [60]. Research has suggested that even though foods with claims may be healthier than their regular counterparts, approximately 25% of the products using health-related claims do not meet the criteria of different profiling schemes [76,77,78]. Although conditions of use for authorised claims may aid in ensuring that clearly unhealthy products cannot bear these claims, this again creates a challenging situation for consumers: even though the specific ingredient highlighted in the claim may be beneficial, is the product overall healthy and thus a good addition to the diet?

Whereas EFSA adopted a scientific opinion on establishing nutrient profiles already in 2008 and consultations with MS followed, this controversial topic ended up being postponed [60,79]. As a result, Article 4 of the NHCR has not yet been dealt with by the regulators. Even though nutrient profiling and front-of-pack nutrition labelling may seem like two separate topics in food policy, the increased attention for front-of-pack nutrition labelling with the development of NutriScore has stimulated the discussion on nutrient profiling and labelling of healthy foods [80]. The new Farm to Fork Strategy, published in May 2020 in the context of the EU’s Green Deal, puts emphasis on front-of-pack labelling for healthy and sustainable foods [81]. These initiatives seem to be closely aligned with developments in national and international dietary recommendations, that have moved from nutrient-based advice to more food based dietary recommendations [82]. This move from focusing on one ingredient for its functionality or health effect to the overall effects of foods is also reflected in scientific research into nutrition: whereas previously, research focused on studying the effects of nutrients in deficiency-related diseases, currently a broader range of effects of the diet is studied [83,84]. The development of nutrient profiles and/or front-of-pack labelling to evaluate overall healthiness of products may help to provide better context for claims to users. Allowing only claims on products that have a positive overall profile or which carry a positive front-of-pack label could aid consumers in selecting healthier products. This could stimulate understanding that even though claims show relevant information about only one ingredient [85], there is more to healthy eating than just focusing on that one ingredient.

The development and use of nutrient profiles and/or front-of-pack nutrition labelling is however immediately complicated by their link to the nutrition and health claim regulation and food information in general. Voluntary food information about the nutritional content of a food product—such as a front-of-pack label—may be provided, as long as this information is not misleading, ambiguous nor confusing and where appropriate, is based on relevant scientific data (Article 36 FIC) [5]. As any form of communicating beneficial ingredients of a food towards consumers is seen as a nutrition claim, also these labelling schemes—being either non-interpretive (reference intake labels) or evaluative (traffic light labels), and providing nutrient-specific or summarised information (healthy choice logos)—will need to be compliant with the NHCR [75]. In many different EU MSs, the Commission’s proposal for mandatory harmonised EU wide front-of-pack labelling has resulted in discussing the use of NutriScore [75]. In France and Belgium, its voluntary use is promoted, whilst in other MS the (mis)alignment of NutriScore with national food based dietary guidance is highly debated [86,87]. Still, the effectiveness of NutriScore and other developed nutrient profiling systems and front-of-pack labelling schemes to contribute to healthier purchase decisions and dietary outcomes is debated [88,89,90,91]. So even though nutrient profiling and front-of-pack nutrition labelling used for nutrition and health claims can support in making claims more informative and decrease the risk of confusing consumers about the healthiness of products, scientific consensus needs to be reached on how to develop profiles and labels in good alignment with national dietary advice.

### 3.3. Consumer Understanding of Claims

As described in Section 2.3, all claims found on the different positive lists can be used by food business operators in their communication strategies towards consumers. As long as these claims provide similar meaning to consumers, they can be adjusted from their technical terminology into a more attractive message or graphic representation for marketing purposes. Various studies have examined the actual use of nutrition and health claims by consumers, and specifically studied consumer understanding of these claims [92,93,94,95,96]. Research seems to lack consensus on how purchase decisions and consumption behaviour are affected by claims, (front-of-pack) nutrition information and food information [97,98], even though evidence suggests that labelling can support identifying and selecting products with healthier ingredients (e.g., with less sodium and trans fats) [99,100]. For claims specifically, it has become clear that their use and understanding are influenced by different consumer characteristics, most importantly personal relevance of the described health benefit [101,102,103,104,105]. While the legislation makes a clear-cut distinction between nutrition claims, function-related health claims and disease risk reduction claims, it is unknown whether consumers can make that same distinction between different types of claims, let alone whether these claims differently influence purchasing and consumption behaviour [92]. Grunert (2017) describes how food-related purchasing decisions often follow more habitual decision-making processes. Technical nutritional information such as claims, may not affect these decisions to a large extent: the overall image and credibility of a healthy product is deemed more important [24]. A 2016 survey conducted by Bandara and colleagues however showed that the majority of 90 Sri-Lankan respondents did examine labels to understand whether these products would be suitable for their lifestyle, health status or preferences [106]. The work of Kolodinsky (2012) highlights that information asymmetry is expected to persist, with a food environment that is changing quickly, industry focusing on profit and consumers being unable or even unwilling to understand and use label information to support healthy dietary decisions [107]. Whilst there is some uncertainty on how nutrition and health claims exactly affect purchasing, claims are used by food business operators to allow their potential customer to show the uniqueness of their products [108]. With an increasing number of targeted, health-influencing products becoming available on the market, it remains important to gain further insights into when—and in what type of wording [109]—such claims can be considered a credible, useful and non-misleading source of information.

### 3.4. Measuring Health Effects

Upon publishing the list of authorised health claims in 2012, a public and scientific debate followed on the limited number of claims authorised: a range of stakeholders felt that the positive list did not do justice on what is known about the health benefits of nutrition [47]. Evidence has only been considered sufficient for substantiating health effects of nutrition when the effect could be attributed to a well-characterised ingredient, when the effect in itself was deemed a beneficial physiological effect (and not a medicinal effect) and when a cause-and-effect relationship was supported, in the case of newly developed claims with two human intervention studies [38]. Whereas some discussions revolved around the set-up of the full procedure, or the level and type of evidence needed to substantiate the health effect, the main issues discussed in the field of nutritional science is (a) the need to reduce the effect of nutrition to one effect of one compound, and (b) to link this effect to health enhancement without it being associated to disease prevention.

Many insights have been gained into the essential role that nutrients play in physiological functions because of occurrence of diseases associated with deficiencies of these same nutrients [83,84,110]. Health benefits of these nutrients can therefore also be measured directly. This type of evidence has supported many health claim applications of Article 13.1 claims, claims that are based on general scientific consensus. However, most nutrients elicit multi-target, more subtle effects, and these substances often do not function in isolation [21,111]. It therefore has been called into question to what extent the currently used pharmaceutical approach to appraise health effects of nutrients reflects actual effects of food ingredients and nutrition on health maintenance and enhancement [21]. This specifically becomes relevant when reflecting on how we define health: such direct biomarkers as study outcomes may reflect health according to the static 1948 definition of the WHO (health as a state of complete physical, mental and social well-being), but may be less appropriate in showing the ability to adapt, a dynamic approach to adapt to circumstances [112,113]. At the same time however, it is this ability to adapt that has been described as a better definition of health: when a biological system is more resilient, it would be more easily able to adapt to circumstances and therefore be considered more healthy [112,113]. Therefore, focusing on resilience as redefined concept of health would better reflects how (chronic) diseases develop and progress, studying a dynamic continuum instead of limiting health and disease to a black and white, yes or no situation [21,114,115,116]. Even though health claim applications so far have not yet made use of resilience measurements as supporting evidence for authorisation requests, such measurements have been suggested to support the health benefits of whole-grain wheat [117]. Measuring this ability to adapt may also present an interesting angle to identify the health benefits of nutrition on a more personal level, allowing for moving towards more personalised and tailored approaches to nutrition that may be supported by claims [118,119]. Before the resilience concept can be used for the authorisation of health claims, however, scientific validation of models which reflect this ability to adapt is key: EFSA’s experts can only critically review scientific opinions when sufficient well-designed and executed studies are supporting the putative relationship between the consumption of a food or ingredient and their suggested health effects.

### 3.5. Enforcement

The NHCR should contribute to ensuring that no false and misleading claims would be found on foods anymore. Like other food legislation however, it is known that regulations do not automatically result in all parties adhering to such legislative requirements [120]. In practice, misleading claims are still reported in online as well as offline sales of foods [121,122]. As with any EU legislation, MSs are responsible for enforcing the NHCR within their jurisdictions. Various MSs provide clear information on how their competent authorities interpret the regulation, by for example providing guidance documents or by making agreements with self-regulating bodies [72,109]. With the aim of the regulation being to protect consumers from misleading information, food law enforcement priorities of these competent authorities may be more focused on protecting consumers from unsafe products instead of reviewing whether nutrition and health claims are used correctly. Such enforcement priorities and strategies are shown to differ between EU MS [123]. Even enforcement actions following incorrect usage of claims takes different forms, ranging from giving advice how to adjust claims to fining the wrong use of claims [72,124]. This affects the extent to which an actual EU-wide level playing field can be created for foods with claims—especially when considering the challenging online market of food sales [125,126]. Therefore, whereas it is important to explore interpretation differences between MSs, to allow for creating actual harmonised legislation throughout the EU, it is essential to prioritise enforcement per se in Member States themselves. Although the risks associated with false claims may be less than the risks associated with exposing consumers to unsafe food, citizens should be able to rely on the enforcement of such a regulated matter. Such controls, or regulatory oversight, have been described as key in ensuring that the regulation in itself can be effectively stimulating the development of a level playing field and adequately contribute to consumer protection and trust [127,128,129].

## 4. Conclusions and Future Recommendations to Support the Use of Well-Regulated Nutrition and Health Claims

This paper analyses how the usage of the EU nutrition and health claim regulation has developed over the last 15 years. This interdisciplinary contribution highlights that to ensure this regulation will stay effective in protecting consumers from false and misleading claims in the next 15 years, it continues to be necessary to address both scientifically and politically the substantiation requirements for claims in relation to botanicals as well as measuring plural and more subtle effects of nutrition. Developments in front-of-pack nutrition labelling should be used to allow the use of claims on only healthy products, and the effects of claims on consumers should continue to be investigated further. Finally, also enforcement strategies should be continuously updated, especially with new challenges arising in protecting consumers from false and misleading claims. Next to the development of new claims related to other product characteristics and credence attributes (such as sustainability and animal welfare), also fake transparency related to nutrition and health should be dealt with proactively: claiming product characteristics that are logic or obvious (‘fat-free strawberries’ or ‘gluten-free water’) should have no place on the EU market.

As highlighted in Section 2, most of the approximately 300 authorised claims were authorised prior to 2017 and are Article 13.1 claims: claims that can be based on different types of evidence versus those claims that are based upon newly developed scientific insights [46]. Most importantly, this section shows that various claims have been pending risk management decisions for a long period of time already: both botanical claims as well as Article 14.1(b) claims targeting infants and young children have awaited authorisation decisions for at least 10 years already. These on hold claims can only be dealt with when political decisions have been made about (a) the type of evidence needed to substantiate health effects of botanicals in foods; and (b) the acceptability of using health claims on foods for infants and young children (up to three years of age). As put forward in Section 3, however, this is not the only issue of the current use and authorisation of nutrition and health claims. To ensure that the regulation can become more effective in protecting consumers from false and misleading claims—and as important, ensure that it can stay effective also in the future—it is essential to ensure that also the other challenges will be addressed. The first two issues described in Section 3, the botanical claims and the lack of nutrient profiles, are challenges that have been identified in the NHCR REFIT evaluation in 2020 already [60]. The Commission needs to take subsequent steps to ensure that these topics are appropriately dealt with. A decision needs to be made whether botanical claims on foods should be assessed similarly to other food products, or whether a separate category needs to be developed for (botanical) claims based on traditional use evidence. Nutrient profiles and specifically, (mandatory) harmonised front-of-pack labelling is one of the agenda points listed in the Commission’s Farm to Fork Strategy. These are important steps in making the NHCR future-proof. Both aspects can be expected to further influence consumer understanding of health claims—the third challenge identified in Section 3. Consumer understanding of claims is an issue that needs to be monitored carefully as the main purpose of the NHCR is to protect consumers from being misled.

This interdisciplinary contribution has aimed to bring together different perspectives that affect the development and use of legislation dealing with nutrition and health information of foods provided to consumers. With its focus on regulatory affairs and nutrition sciences, the paper reflects on the past 15 years of regulating such claims in the EU and provides multi-level insights and suggestions for moving forward in the years to come. Even though it touches upon different disciplines, this study is limited in providing detailed insights into academic disciplines related to consumer understanding and use of claims and front-of-pack labelling schemes, as well as enforcement. Voluntary information provision for foods and food ingredients is a highly dynamic, interdisciplinary and fast-developing field, that is not only affected by health considerations or food policy, but also by marketing, food design as well as sustainability and animal welfare considerations. It will therefore remain essential to continue studying the relevant disciplines both into depth and empirically, to critically review whether the suggestions proposed in this paper indeed contribute to further increasing consumer protection and at the same time, stimulate innovation in the field of nutrition and health claims.

To ensure that the Regulation also stimulates innovation in the EU—one of the predefined goals of the NHCR—there are also two other challenges that need to be paid attention to [127]. First, related to profiling foods as more or less healthy, it is essential that research into establishing health effects of foods is in line with the latest definition of health: the ability to adapt. So far, nutrition research has mainly relied on the use of a more pharmaceutical approach to establishing health effects of nutrients, whilst methods to analyse the ability to adapt (or reflexivity) may provide better insights into the pleiotropic and subtle effects associated with food intake. Secondly, only with online and offline enforcement, can the NHCR live up to its promises of protecting consumers from misleading information. It is essential that consumers can trust that all claims displayed on labels, in advertisements and on (social) media are trustworthy. Tackling these political and scientific substantiation questions for health claims, together with continuously analysing the understanding and usage of claims by consumers and operators will ensure that the NHCR will stay effective, today and in the future.

## Figures and Tables

**Table 1:** Authorised nutrition and health claims in the EU.

Claim Type	Authorised Claims	Authorised in
Nutrition claim	30	2006–2012
Article 13.1 claim	229 ^1^	2012–2016
Article 13.5 claim	11 ^2^	2009–2017
Article 14.1(a) claim	14	2009–2014
Article 14.1(b) claim	12	2009–2016

^1^ Positive list in Annex of Regulation (EU) No 432/2012. ^2^ Grouping the two entries related to the authorised health claim on cocoa flavanols, including the authorised health claim on water-soluble tomato concentrate that is not defined in the positive list in Regulation 432/2012, but is laid down in Commission Decision 2009/980/EU.

**Table 2:** Health claims pending risk management decisions.

Type	Pending	Scientific Opinion ^1^
Article 13.1 claim	2073 ^2^	n/a
Article 13.5 claim	15	6 positive opinions: - No additional comments, cause-and-effect relationship established (2) - Comments upon safety in response to issued opinion (1) - Positive effects associated with more specific ingredient (1) - Claim has already been assessed with favourable outcome (2). 9 negative opinions: - Cause-and-effect relationship not established (6) - Evidence provided insufficient to establish cause-and-effect relationship (2) - Claimed effect does not refer to any specific health effect measurable in vivo (1).
Article 14.1(a) claim	4	1 positive opinion: - No additional comments, cause-and-effect relationship established (1) 3 negative opinions: - Cause-and-effect relationship not established (1) - Evidence provided insufficient to establish cause-and-effect relationship (2)
Article 14.1(b) claim	25	24 positive opinions: - No additional comments, cause-and-effect relationship established (1) - Claims target infants and young children (up to 3 years), and deal with claims that have been assessed positively for other age groups already (21) - Claims target children from infant up to 18 years of age but had been targeted to infants and young children by applicant (2).^3^ 1 negative opinion: - Evidence provided does not establish a beenfit (1).

^1^ The scientific evidence of claims listed in this table has been reviewed. The risk manager has however not yet issued a decision on whether the claim will be authorised or rejected. ^2^ Botanical claims for which finalisation is pending listed in the ‘on hold’ list, encompassing 2133 claim ID numbers [28]. ^3^ In response to one of these scientific opinions, the European Responsible Nutrition Alliance suggested to allow one general claim that ‘vitamins and minerals are required for normal growth and development of children’ for the target group of children from 3 to 18 years, for essential nutrients.

## Data Availability

Not applicable.
